# The influence of physical and mental workload on the safe behavior of employees in the automobile industry

**DOI:** 10.1016/j.heliyon.2022.e11034

**Published:** 2022-10-11

**Authors:** Fateme Jame Chenarboo, Reza Hekmatshoar, Majid Fallahi

**Affiliations:** a Department of Occupational Health Engineering, Student Research Committee, Sabzevar University of Medical Sciences, Sabzevar, Iran; b Department of Occupational Health Engineering, Faculty of Health, Sabzevar University of Medical Sciences, Sabzevar, Iran; c Department of Occupational Health Engineering, Faculty of Health, Non Communicable Disease Research Center, Sabzevar University of Medical Sciences, Sabzevar, Iran

**Keywords:** Physical workload, Mental workload, Safe behavior

## Abstract

This study aimed to investigate the influence of physical and mental workload on safe behavior of employees in the automobile industry. The 150 workers of the two industries of machining and foundry of an automobile parts manufacturer participated in this correlational study. Safety behavior, NASA-TLX, and Borg scale questionnaires were used to collect data. Independent t-test, analysis of variance and Pearson correlation coefficient applied to the analysis of data. The NASA-TLX showed that the dimensions of physical and mental demand had the highest score and the performance had the lowest score. Excessive physical pressure was also reported among workers. The Score of safety observance, safety participation, and safety behavior were at a moderate level. There was a significant difference in the physical workload of employees who had an accident and did not have an accident (P = 0.001). The results showed that if the same mental workload had been imposed on workers and simultaneously more physical workload had been experienced, the probability of an accident increased. The overall mental workload and physical pressure among workers were reported at a high level. Safe behaviors were moderate among employees. Therefore, the implementation of effective intervention programs to adjust workload, participatory ergonomics, provide workload balance to improve job satisfaction, eliminate inappropriate working conditions and increase the number of operators, management programs such as job rotation between Machining and Foundry and other workshops, increase rest time and creation of a strong teamwork safety climate can reduce physical and mental workload and prevent accident among workers, improve their performance and wellbeing.

## 1 Introduction

Injuries and accidents in the workplace remain a serious safety concern worldwide [1, 2]. According to the International Labor Organization, approximately 7600 people die every day as a result of occupational disease or workplace accidents [3]. Unsafe behaviors, which are performed by humans and always occur, are the primary cause of accidents. Heinrich et al. (1980) concluded that 88% of accidents are due to unsafe behaviors [4]. Safety accidents in various industries not only lead to personal injury but also have high economic costs [5, 6]. Haslem et al., after analyzing 100 accidents in the construction industry, found that 70% of these accidents are related to human factors, especially the unsafe behavior of employees [7]. Therefore, the reduction in the occurrence of occupational injuries and accidents largely depends on the promotion and improvement of employee safety behavior. For example, Nahrng et al. [8] consider the perceived level of workplace hazards as one of the types of job barriers (e.g., stress), which requires constant physical and/or mental effort. As a result, it leaves limited personal resources for managing safety behaviors [9]. If managers understand the factors that affect employees' job stress, they can prevent many accidents [10]. Understanding the role of job needs in workplace safety is important in safety initiatives that can target the physical and psychological factors of work [11].

To understand these needs and promote safe behavior, it is necessary to pay attention to the workload imposed on workers. There are several methods of subjectively assessing the workload for workers to assess their perception of physical and psychological needs independently. The Borg Scale measures the perceived difficulty of performing physical work [12], and the NASA Task Load Index (TLX) is a common tool for measuring the operator’s MWL about cognitive tasks [13, 14]. The NASA-TLX rating method has shown sensitivity to different levels of mental and physical workload while performing simultaneous work [15]. In general, the simple scales used to assess PWL and MWL, respectively, include the Borg (CR10) Scale and the NASA-TLX. These two methods are sensitive to changing needs in their respective fields without being affected by simultaneous tasks. Limitations to the assessment of MWL arise when individuals are required to make a combined assessment of physical needs [16].

A growing body of prior literature addresses a variety of factors that influence safety behaviors, including effective training [17], regulatory behavior [18], nationality [19], safety climate [20, 21, 22], attitudes [23], leadership [24], risk perception [25], job stress and emotional intelligence [26] organizational climate and individual differences [27]. However, the scientific study of the influence of physical and MWL on the safe behaviors of employees has received less attention. In some occupations, such as the manufacturing, firefighting, and assembly industries, in addition to performing physical tasks, workers also perform mental activities at the same time [28]. In the manufacturing industry, due to the complexity of technology, it is necessary to pay attention to the proper design of the human-machine system to increase its effectiveness. Therefore, it is important to evaluate and pay attention to the mental load of the workers [29]. In the real world, many jobs require the simultaneous processing of mental information along with the physical activity. Therefore, the effect of PWL on workers' mental performance and sources of attention cannot be ignored [30]. In the manufacturing industry, paying too much attention to the cost and efficiency of the system imposes a lot of work pressure on workers. In these industries, the complexity of the process and equipment has a great impact on the mental and physical burden of workers. Improper design of processes and equipment can impose a lot of mental and PWL, which in turn will reduce job performance and satisfaction [31].

In this industry, unfortunately, the lack of efficient occupational safety and health management system was evident. In the field inspection and based on the accidents that occurred in this industry, it was observed that one of the most important factors influencing the occurrence of accidents was workload (mental and physical) and subsequent unsafe behaviors. Therefore, after justifying the employer and the occupational safety and health management system, As a result of discussions with all workers, the focus was on modifying the physical and MWL, as well as promoting safe behaviors. Therefore, in this study, an attempt is made to investigate the influence of MWL and PWL on safety behaviors. According to the results that will be obtained, it is possible to take a step towards improving safety behaviors in the workplace by providing suggestions to improve the safety conditions of work and human resources according to the studied factors.

## 2 Literature review

### 2.1 Workload

“Workload is a term that represents the cost of accomplishing mission requirements for the human operator” [32]. The workload is physical and mental and both are always related to each other and when a person performs a specific task, they cannot be completely separated [33]. MWL assessment methods are commonly used to assess work-related mental and physical workload. There is evidence that existing assessment tools can also be used to assess tasks that involve both mental and physical needs, although multidimensional tools can also estimate the total workload [16]. Workload has long been considered an important and influential factor in individual performance in complex systems. The Workload is divided into two groups: physical workload (PWL) and mental workload (MWL) [34]. The Workload consists of a group of elements such as environment, community, motivation, and other factors that affect the operator’s ability to perform the task [35]. Thus, when the frequency or difficulty of the tasks necessary to accomplish a goal increases, or when the time allotted for completing the tasks decreases, the workload increases [36].

Workload evaluation is one of the most important components of system analysis and design. A subjective account of a person’s perception of physical or mental work, in general, shows the nature and nature of the task and its need for physical and cognitive resources [37]. People are often expected to perform physically demanding tasks at the same time as their cognitive responsibilities, with the continued implementation of technology. According to the needs and expectations that are imposed on the individual while performing a complex task, the impact and interaction of physical and mental activities determine the critical workload levels of work [16].

### 2.2 Safety behavior

The term safety behavior is the activity performed by individuals in an organization related to safety [38]. Physical and psychosocial safety behaviors result from the activities of employees that support and protect the physical and psychological safety of the work environment or create environments that lead to safety support [39]. Physical safety behaviors include the use of personal protective equipment, safe work with machinery, and active and preventive participation in safety recommendations [40]. Psychosocial safety behaviors include changing work habits to reduce work stress or initiate actions, such as reporting an incident or event [39]. Valuing these behaviors creates an environment that supports physical and social safety [39, 41]. In terms of safety compliance, employees are expected to follow the organization’s specific rules, regulations, and procedures, as opposed to safety participation, which focuses on employee participation in safety-related activities [40, 42]. To prevent accidents and injuries, organizations depend on the safety and participation of their employees. However, the relationship is different. Observance of safety has a direct effect on accidents because it is related to the observance or non-observance of organizational policy [40]. In contrast, participation in safety is more indirect because participation or non-participation does not necessarily lead to an accident. Rather, participation can help reduce accidents by improving the work environment through safety training or accident reporting to improve safety at work [40, 42]. Both aspects of physical and psychosocial safety climate directly affect participation in safety behavior. Bronkhorst (2015) found that the independence of job resources and supportive environment, as well as psychological climate and physical safety, have a positive relationship with physical and psychosocial safety behavior. For example, when employees face a high workload, they use less safety equipment or report fewer accidents [39].

Employees who are under a lot of work pressure due to aggression or violence (psychological safety) are less likely to use safety equipment (physical safety) or start and report an accident. Mullen [43] found that performance stress is an important factor influencing safety behavior in the workplace because people under stress tend to value performance over safety (sacrificing safety for performance). Other previous research has shown a negative relationship between job needs and safety behavior [8, 43]. Therefore, we argue that increasing job demand will lead to a decrease in physical and psychosocial safety behavior among employees [39].

## 3 Materials and methods

### 3.1 Research strategy and sampling techniques

**Figure 1:**
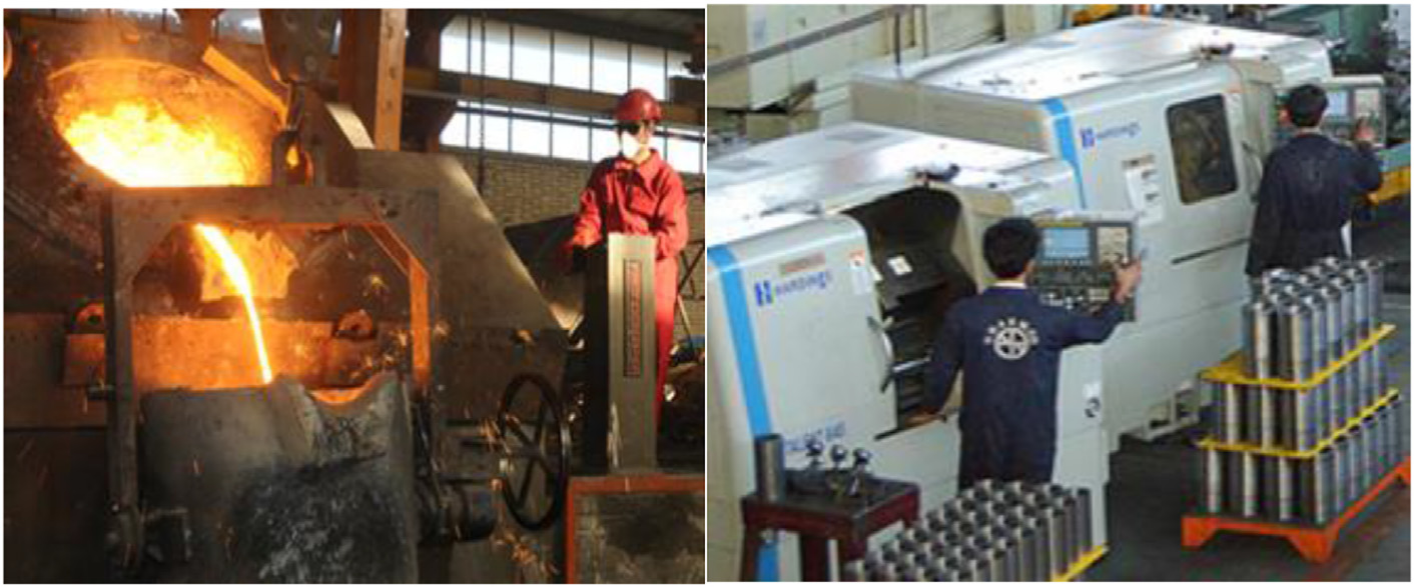
Machining (Right) and Foundry (Left) site.

The present study is a descriptive-analytical cross-sectional study. The study population was all the 150 workers in two industrial units of machining and foundry (Figure 1) in automobile industry. The response rate was 88%. Inclusion criteria were having at least 2 years of work experience, not suffering from certain diseases, and not have serious physical or mental health problems. These criteria were determined based on the health records of the workers. The ethical consent form was given to the participants and the purpose of the study was explained to them. Due to the anonymity of the questionnaires and the preservation of privacy and the absence of aggressive samples, there were no complications for the workers. 18 workers were excluded from the study due to unwillingness to cooperate and lack of inclusion criteria, and 132 people were examined. In the next stage, after the coordination that was done in advance, on a specific day, time and place, the safety behavior questionnaire was given to the workers at the end of the work shift and the questionnaires were completed by the individuals themselves in a semi-supervisory manner. Employees were also asked if they had a work accident. Then, on another day and time, at the end of the work shift, the NASA-TLX Questionnaire and the Borg Scale Questionnaire were provided to the workers.

### 3.2 The questionnaires

#### 3.2.1 NASA-TLX method

The NASA-TLX questionnaire was developed by the Human Performance Group at NASA Research Center [13]. This method has been used in many studies of human-computer interaction as a method of measuring MWL. A multidimensional rating method contains six subscales: mental demand, physical demand, time demand, performance, effort, and frustration. The first three subscales are related to the participant’s needs and the last three are related to the participant’s interaction with the task. The numerical rating for each subscale is determined by the participant on a line divided into 20 equal distances, which becomes the 0–100 rating scale. The line for each subscale on each side of the line is identified by unipolar descriptors (for example, high and low). The raw workload score is calculated by summing the rates provided for each of the six subscales and dividing it by six [44]. The most common total workload score is calculated based on the weighted average rating of the subscales. The weight of the subscales is determined by the participant’s assessment of what each factor contributes to the workload. After completing the initial rating, 15 pair-pair comparisons of the subscales are shown to the individual, and the participant selects a subscale of 15 pairs that he or she considers important in the task at hand. The weight of each subscale is 15 times the number of selected items. The total workload is calculated by multiplying each dimension of the weighting by the weight obtained from it under the scale and has a range of 0–100. It is more diagnostic and detailed than the one-dimensional scale [45]. In this study, if the score was less than 40, 40–60, and more than 60, it was evaluated as low, medium, and high for each dimension, respectively.

#### 3.2.2 Safety behavior questionnaire

The safety behavior Questionnaire has been prepared by Mehdi Nia et al. (2016). The reason for using this questionnaire was its validity and reliability were carried out among the 294 workers in manufacturing and process industries. The questionnaire consists of 23 questions, 12 of which are in the field of safety compliance and 11 questions in the field of safety participation, which are graded in five grades (5 always, 4 often, 3 sometimes, 2 rarely and 1 never). In this questionnaire, the higher score indicates safer behavior. The validity and reliability of this questionnaire have been measured in studies. The average agreement ratio about simplicity, clarity, and relevance of all questionnaire questions was equal to 0.91 and concerning necessity was equal to 0.95. The value of ICC (Intraclass Correlation Coefficient) index was equal to 0.752 (p = 0.009) and Pearson correlation coefficient between test results and retest was 0.619 (p = 0.02). Cronbach’s alpha coefficient was 0.902 [46]. In this study, the levels of safety behavior and its dimensions for scores less than 2, 2–3.5, and more than 3.5 were considered equal to low, medium, and high, respectively.

#### 3.2.3 Borg questionnaire (CR10)

This questionnaire is used to subjectively assess the PWL (or perceived difficulty) that is widely used to assess physical activity [12, 47]. The Borg scale is a numerical scale from 0 (not at all) to 10 (very, very high) that consists of several verbal anchors to increase the usability of the scale by individuals. Refer to source [12] for a detailed description of the scale and how it is implemented.

### 3.3 Method and procedure for data analysis

The data were recorded in SPSS software (version 22) and descriptive results were extracted using descriptive methods including frequency distribution tables, graphs, and descriptive indicators, and then the variables were normalized using the Kolmogorov-Smirnov test. Quantitatively examined and after confirming the normality of the data, to compare the two means of independent t-test, to compare several means of the analysis of variance, to compare the mean for binary observations of paired t-test, for multiple observations of repeated measures test and Pearson correlation coefficient, were used to investigate the relationship between quantitative variables. If the quantitative variables did not have a normal distribution, the corresponding non-parametric Mann-Whitney, Kruskal-Wallis, Wilcoxon, Friedman, and Spearman correlation tests were used. The confidence level in all tests was 95%.

## 4 Results

The results of the NASA-TLX Questionnaire regarding the mental assessment of employees' MWL showed that the dimensions of physical demand and mental demand have the highest score and the performance dimension has the lowest score. Also, the results of the Borg questionnaire, showed that physical pressure was at a high level (Table1).

**Table 1:** Mean and standard deviation of NASA-TLX dimensions (N = 132).

Dimension	Mean	Std. Deviation	Level
Mental demand	77.88	26.61	high
Physical demand	87.61	13.11	high
Temporal demand	76.33	23.93	high
Performance	34.70	24.74	low
Effort	76.29	20.80	high
Frustration	71.70	21.75	high
Overall MWL	68.85	11.23	high
Physical pressure	17.64	2.86	high

Also, the results of the dimensions of safety behavior have been illustrated in Table2. It was determined that the score of safety observance, safety participation, and safety behavior is moderate among employees.

**Table 2:** Mean and standard deviation of dimensions of safety behavior in employees (N = 132).

Dimension	Minimum	Maximum	Mean	Std. Deviation	Level
safety observance	1.28	5.00	3.50	0.76	Moderate
safety participation	1.36	4.80	3.10	0.72	Moderate
safety behavior	1.55	4.70	3.29	0.64	Moderate

The results of safety behavior dimensions have been illustrated in Table 3. A significant difference was observed between the two groups (employees who had an accident and employees who did not have an accident) in safety observance, safety participation, and safety behavior dimensions (p < 0.05) (Table4).

**Table 3:** Mean and SD of three dimensions of safety behavior.

Accident		Safety observance	Safety participation	Safety behavior
Yes (N = 59)	Mean	2.05	2.00	2.01
	Std. Deviation	0.39	0.37	0.34
No (N = 73)	Mean	2.32	2.12	2.21
	Std. Deviation	0.52	0.37	0.44
Total	Mean	2.20	2.06	2.12
	Std. Deviation	0.49	0.37	0.41

**Table 4:** Results of Analysis of Variance in the dimensions of safety behavior among accident (N = 59) and non-accident (N = 73) employees.

Dimension		Sum of Squares	df	Mean Square	F	Sig.
Safety observance ∗ accident	Between Groups	2.52	1	2.52	11.31	0.001
	Within Groups	28.95	130	0.22		
	Total	31.47	131			
Safety participation ∗ accident	Between Groups	0.49	1	0.49	3.60	0.048
	Within Groups	Within Groups	130	0.13		
	Total	Total	131			
Safety behavior ∗ accident	Between Groups	1.33	1	1.33	8.07	0.005
	Within Groups	Within Groups	130	0.16		
	Total	Total	131			

Mean and SD of overall MWL and physical pressure among employees who had experienced accidents compared to non-accident employees have been shown in Table 5. According to the results of Analysis of Variance, a significant difference was observed in the PWL (Borg scale results) of employees who had an accident and did not have an accident. But this difference was not significant for the six dimensions of NASA-TLX and weighted workload in the two groups (P > 0.05) (Table 6).

**Table 5:** Mean and SD of overall MWL and physical pressure among accident (N = 59) and non-accident (N = 73) employees.

Accident		Overall MWL	Physical Pressure
Yes	Mean	68.93	19.69
	Std. Deviation	11.95	2.26
No	Mean	68.78	15.33
	Std. Deviation	10.69	2.56

**Table 6:** Results of overall MWL and PWL analysis of variance among accident (N = 59) and non-accident (N = 73) employees.

Workload		Sum of Squares	df	Mean Square	F	Sig.
Overall MWL ∗ Accident	Between Groups	0.67	1	0.67	0.005	0.942
	Within Groups	16,526.70	130	127.12		
	Total	16,527.37	131			
Physical pressure ∗ Accident	Between Groups	4.37	1	4.37	0.738	0.003
	Within Groups	770.61	130	5.92		
	Total	774.99	131			

The results of the analysis of variance showed that there is a significant relationship between the dimension of mental demand and dimensions of safety behaviors (Table 7) and also the dimension of time demand and dimensions of safety behaviors (Table 8).

**Table 7:** Results of analysis of variance for mental demand and three dimensions of the safety behavior questionnaire.

Dimension			df	Mean Square	F	Sig.
Safety observance	Between Groups	2.619	2	1.310	5.854	0.004
	Within Groups	28.858	129	0.224		
	Total	31.477	131			
Safety participation	Between Groups	.445	2	0.223	1.600	0.206
	Within Groups	17.941	129	0.139		
	Total	18.386	131			
Safety behavior	Between Groups	1.353	2	0.676	4.066	0.019
	Within Groups	21.458	129	0.166		
	Total	22.811	131

**Table 8:** Results of analysis of variance for time demand and three dimensions of safety behavior questionnaire.

Dimension			df	Mean Square	F	Sig.
Safety observance	Between Groups	1.451	2	0.725	3.116	0.048
	Within Groups	30.027	129	0.233		
	Total	31.477	131			
Safety participation	Between Groups	1.142	2	0.571	4.273	0.016
	Within Groups	17.244	129	0.134		
	Total	18.386	131			
Safety behavior	Between Groups	1.940	2	0.970	5.995	0.003
	Within Groups	20.871	129	0.162		
	Total	22.811	131			

## 5 Discussion

This study aimed to investigate the influence of PWL and MWL on the safe behaviors of employees in automobile industry. The results of this study showed that the total physical pressure and MWL imposed on employees are high. The results of the study by Rezaei et al. [48] showed that mental and PWL are effective on accident statistics. Physical stress and high MWL imposed on employees together can lead to an increase in human error and result in accidents. In the present study, the total workload and physical pressure were reported at a high level. In a study by Mazloumi, et al. [49] the level of MWL reported by the assembly line workers of a car factory was high; which is consistent with the present study.

In the study of Khandan et al. [50], among the different dimensions of the workload from the perspective of workers in a heavy metal parts industry, two dimensions of time load and physical load obtained the highest score, which in the present study, three dimensions of physical, temporal and mental had the highest score. In the study of Taheri et al. [51], the results showed that there was a significant difference between the mean of mental demand and time demand among people who were needled, compared to people who were not needled. In the present study, the results showed that there was a significant difference between the mean score of mental demand and time demand between employees who had an accident and employees who did not have an accident and there was no significant relationship with other dimensions of NASA-TLX. In a study by Oah S, et al. (2018) a positive significant correlation was observed between worker workload and accident experience [52].

In this study, the Pearson correlation coefficient between the average physical demand subscale of NASA-TLX and the average physical pressure (Borg scale) was 0.61. The study findings of Nasirizad Moghadam et al (2021) revealed a significant relationship between physical and MWL [53]. The results of a study showed that NASA-TLX scores as well as many NASA TLX subscales are sensitive to changes in the amount of physical and mental work [15]. In one study, by tracking the operational process via video, occupational factors that could lead to high workload were tracked and used in designing the manufacturing process to reduce mental and PWL and improve optimal production efficiency [31].

Regarding dimensions of safety behavior, the scope of safety participation had the lowest score. In order to increase and enhance employee safety participation, it is recommended to use appropriate incentive schemes and an effective plan to establish a safety culture and improve safety behaviors by the occupational safety and health officer to improve the safety level of the organization. The role of human factors in the occurrence of accidents in the industry is undeniable. Therefore, it is possible to reduce the effect of this factor by using educational intervention and increasing awareness and behavior modification among employees, and this intervention can lead to a reduction in unsafe behavior and, consequently, a reduction in occupational accidents. Although, Oah S, et al. (2018) suggest that conducting active risk management provides a positive safety climate at the organizational level and may decrease employees' risk perceptions [52]. The study results showed that workers with a history of accidents reported significantly less safety behavior. There was also a significant relationship between PWL and accidents. The result of a study among health workers showed that there was a negative significant relationship between work pressures with safety behavior [54].

Adopting an “ideal” approach in the industry is effective by implementing measures that moderate the burden of physical and mental work. And the important thing is for employees to identify problems and, with the help of managers, come up with ideas for improvement [55]. To improve the organization’s public safety performance, behavioral safety training based on information sharing and encouraging colleagues to talk about safety issues should be emphasized. It is also necessary to discuss preventive safety issues among employees who need to correct and improve safe behaviors. The formed health and safety groups should devote their efforts to developing persuasive messages by targeting important variables and objective safety interventions to improve or change safety behaviors. Practical work in the field of safety intervention should be based on the design and implementation of programs designed to change the main variables, especially social norms, which in turn are expected to have beneficial effects on personal factors and individual behaviors [56]. Regarding the results of the study by Schwartz et al. (2020), to reduce the amount of PWL and MWL experienced by employees in such industry, it seems important to consider strategies that reduce ergonomic workload and the burden of cognitive work. Expansion of this work should include examining the relationship between ergonomic workload and stress in employees of different workshops in the industry. In addition, the careful study and evaluation of the workload of different occupations, and the design and implementation of interventions that can be implemented to ensure the reduction of PWL and MWL in these occupations will be of particular importance [57].

## 6 Limitations and future studies

A limitation of this study is that since this study was conducted in two workshops of an automobile industry, its results cannot be generalized to other industrial occupations. Also, only men were active in this industry, and it is recommended that a wider population, as well as women, be participated in future studies. Another limitation of this study is the use of self-report scales. In future studies, various criteria of safety behavior, such as the supervisor ratings of safety behavior, can be used to overcome potential bias. Finally, the present study carried out cross-sectional and it is recommended that future studies be designed to prospective cohorts in different industries that have and do not have occupational health and safety management system. Furthermore, the future researcher can use other methods to evaluate the physical and MWL and safe behaviors.

## 7 Conclusion

Regarding the findings of the study, the total workload and physical stress were reported to be high among workers. Also, high-level exposure to five dimensions of mental demand, physical demand, time demand, effort, and frustration were reported by employees. The three dimensions of safe behaviors, safety observance, and safety participation were moderate among employees. Employees who had an occupational accident endured more physical stress in addition to MWL compared to employees who did not have an accident. Therefore, it can be acknowledged that in activities that impose on employees both mental and physical needs, if it is the same mental demand, high physical pressure can make employees more prone to accidents. In addition, according to the results of the NASA-TLX, the dimension of physical need had the highest score compared to other dimensions, which seems to confirm this. Therefore, the implementation of effective intervention programs to adjust workload, participatory ergonomics [58], workload balance to improve job satisfaction among workers [59], eliminate inappropriate working conditions and increase the number of operators, and management programs such as job rotation between Machining and Foundry with other workshops, increase rest time and creation of a strong teamwork safety climate can reduce physical and MWL and prevent accident among workers. In addition, providing educational programs for workers about factors to improve how they manage their workloads such as time management and safety behaviors may be important and useful strategies to improve the performance of workers and occupational wellbeing.

## Declarations

### Author contribution statement

Fateme Jame Chenarboo; Reza Hekmatshoar; Majid Fallahi: Conceived and designed the experiments; Performed the experiments; Analyzed and interpreted the data; Contributed reagents, materials, analysis tools or data; Wrote the paper.

### Funding statement

This work was supported by Student Research Committee of the Deputy of Research and Technology of Sabzevar University of Medical Sciences.

### Data availability statement

Data included in article/supp. material/referenced in article.

### Declaration of interest’s statement

The authors declare no conflict of interest.

### Additional information

No additional information is available for this paper.

### Additional information

No additional information is available for this paper.

## Appendix A Supplementary data

QUESTIONNAIRES QUESTIONNAIRES
