# Supplementary material for: The influence of physical and mental workload on the safe behavior of employees in the automobile industry
Source: Heliyon. 2022 Oct 11;8(10):e11034. doi: 10.1016/j.heliyon.2022.e11034 (PMC9582718; doi:10.1016/j.heliyon.2022.e11034)
Supplement: QUESTIONNAIRES [file mmc1.docx]

**QUESTIONNAIRES**

**English Version: Borg RPE**

**English Version: NASA-TLX**

Persian Version: BORG RPE

مقیاس RPE 6-20 بورگ

| درجه | شرح فشار جسمانی |
| --- | --- |
|  | هیچ نوع فشاری اعمال نمی­شود. |
|  | فوق العاده سبک |
|  | بسیار سبک |
|  | سبک |
|  | کمی سخت |
|  | سخت |
|  | بسیار سخت |
|  | فوق العاده سخت |
|  | فشار حداکثر |

Persian Version: NASA-TLX

- شاخص بار کار ناسا

دوست گرامي؛ پرسشنامه اي كه پيش رو داريد، در مورد روش ذهنی ارزیابی بار کار فکری است. نتايج مفيد اين تحقيق در گرو پاسخ دقيق و صادقانه شماست. بنابراين از شما خواهشمندیم سوالات را با توجه به وظیفه­ای که انجام می­دهید با دقت پا­­سخ دهيد. نیازی به ذکر نام و نام خانوادگي نیست. از اينکه وقت خود را در اختيار ما قرار مي­دهيد، بسيار سپاسگزاريم.

بخش نخست: شرکت کننده محترم با توجه به وظیفه ای که انجام می­دهید و توصیف مربوط به هر بعد، به صورت ذهنی ارزش هر بعد در وظیفه تان را بر روی هر مقیاس با علامت ضربدر ]×[ مشخص کنید.

1. نیاز فکری: وظیفه­ای که انجام می­دهید به چه میزان نیاز فکری احتیاج دارد؟

|  | | | | | | | | | | | | | | | | | | | |
| --- | --- | --- | --- | --- | --- | --- | --- | --- | --- | --- | --- | --- | --- | --- | --- | --- | --- | --- | --- |
|  |  |  |  |  |  |  |  |  |  |  |  |  |  |  |  |  |  |  |  |
|  |  |  |  |  |  |  |  |  |  |  |  |  |  |  |  |  |  |  |  |
| خیلی کم | | | | | | | | | | خیلی زیاد | | | | | | | | | |

2. نیاز فیزیکی: وظیفه­ای که انجام می­دهید به چه میزان نیاز فیزیکی احتیاج دارد؟

|  | | | | | | | | | | | | | | | | | | | |
| --- | --- | --- | --- | --- | --- | --- | --- | --- | --- | --- | --- | --- | --- | --- | --- | --- | --- | --- | --- |
|  |  |  |  |  |  |  |  |  |  |  |  |  |  |  |  |  |  |  |  |
|  |  |  |  |  |  |  |  |  |  |  |  |  |  |  |  |  |  |  |  |
| خیلی کم | | | | | | | | | | خیلی زیاد | | | | | | | | | |

3. نیاز زمانی: وظیفه­ای که انجام می­دهید به چه میزان عجله و شتاب احتیاج دارد؟

|  | | | | | | | | | | | | | | | | | | | |
| --- | --- | --- | --- | --- | --- | --- | --- | --- | --- | --- | --- | --- | --- | --- | --- | --- | --- | --- | --- |
|  |  |  |  |  |  |  |  |  |  |  |  |  |  |  |  |  |  |  |  |
|  |  |  |  |  |  |  |  |  |  |  |  |  |  |  |  |  |  |  |  |
| خیلی کم | | | | | | | | | | خیلی زیاد | | | | | | | | | |

4. کارایی: تا چه میزان در انجام کامل وظیفه­ای که از شما خواسته شده، موفق هستید؟

|  | | | | | | | | | | | | | | | | | | | |
| --- | --- | --- | --- | --- | --- | --- | --- | --- | --- | --- | --- | --- | --- | --- | --- | --- | --- | --- | --- |
|  |  |  |  |  |  |  |  |  |  |  |  |  |  |  |  |  |  |  |  |
|  |  |  |  |  |  |  |  |  |  |  |  |  |  |  |  |  |  |  |  |
| کاملا موفق | | | | | | | | | | کاملا ناموفق | | | | | | | | | |

5. تلاش و کوشش: تا چه میزان باید به سختی کار کنید تا به سطح عملکرد دلخواه خود برسید؟

|  | | | | | | | | | | | | | | | | | | | |
| --- | --- | --- | --- | --- | --- | --- | --- | --- | --- | --- | --- | --- | --- | --- | --- | --- | --- | --- | --- |
|  |  |  |  |  |  |  |  |  |  |  |  |  |  |  |  |  |  |  |  |
|  |  |  |  |  |  |  |  |  |  |  |  |  |  |  |  |  |  |  |  |
| خیلی کم | | | | | | | | | | خیلی زیاد | | | | | | | | | |

6. ناکامی و سرخوردگی: در طول کار چقدر احساس ناامنی، دلسردی، خشم، استرس و رنجش دارید؟

|  | | | | | | | | | | | | | | | | | | | |
| --- | --- | --- | --- | --- | --- | --- | --- | --- | --- | --- | --- | --- | --- | --- | --- | --- | --- | --- | --- |
|  |  |  |  |  |  |  |  |  |  |  |  |  |  |  |  |  |  |  |  |
|  |  |  |  |  |  |  |  |  |  |  |  |  |  |  |  |  |  |  |  |
| خیلی کم | | | | | | | | | | خیلی زیاد | | | | | | | | | |

Persian Version: Safety Behavior Questionnaire

- There is no English version for safety beahviour questionnaire

به نام خدا

حفظ سلامت روان و جان شما گرانمایه ترین اصل در زندگی به شمار می آید.

**همکار گرامی سلام ؛**

**این پرسشنامه پژوهشی با عنوان تاثیر بار کار فکری و جسمانی بر رفتار ایمن کارگران تهیه شده است. همکاری و صبر و حوصله شما در پاسخ دقیق به سوالات مطرح شده موجب دست یابی بهتر و صحیح تر شده و امکان رسیدن به نتایج مطلوب تر را فراهم می­آورد. لذا خواهشمند است با دقت به سوالات پاسخ دهید. به جهت اطمینان خاطر شما پاسخ دهنده گرامی، نیازی به ذکر اسم و فامیل­تان نیست.**

1- سن : ...... سال

2- وضعیت تاهل : مجرد متاهل

3- سطح تحصیلات : زیر دیپلم دیپلم فوق دیپلم کارشناسی

0

4- شغل شما: ریخته گری ماشین کاری

0

5- آیا در یکسال گذشته دچار حادثه شده­اید؟ بله خیر

0

| ردیف | سوال | هرگز | به ندرت | گاهی | اغلب | همیشه |
| --- | --- | --- | --- | --- | --- | --- |
| 1 | به دستورالعمل ها و قوانین ایمنی مربوط به کارم، عمل می­کنم. |  |  |  |  |  |
| 2 | تحت تاثیر همکاران با شرایط محیط کار، قوانین و دستورالعمل های ایمنی را نقص میکنم. |  |  |  |  |  |
| 3 | تحت تاثیر فشار مدیریت، قوانین و دستورالعمل های ایمنی را نقص میکنم. |  |  |  |  |  |
| 4 | تحت تاثیر فشار کار یا به دلیل کمبود وقت، قوانین و دستورالعمل ها ی ایمنی را نادیده میگیرم. |  |  |  |  |  |
| 5 | با نادیده گرفتن بعضی از قوانین، بعضی از کارها را بهتر و سریعتر انجام میدهم. |  |  |  |  |  |
| 6 | کارم را بدون عجله و با سرعت ایمن انجام میدهم. |  |  |  |  |  |
| 7 | هشدار و علایم ایمنی را جدی می­گیرم. |  |  |  |  |  |
| 8 | برای انجام کارم از تمامی وسایل حفاظت فردی و تجهیزات ایمنی مربوطه استفاده می­کنم. |  |  |  |  |  |
| 9 | در انجام کارم از ابزارها و وسایل مناسب و سالم استفاده می کنم. |  |  |  |  |  |
| 10 | به دلیل تسلط و تجربه بالا در کارم، نکات ایمنی را نادیده می­گیرم. |  |  |  |  |  |
| 11 | مسئولیت کارهای خطرناک را می پذیرم و انجام میدهم. |  |  |  |  |  |
| 12 | محیط کارم را تمیز و مرتب نگه میدارم. |  |  |  |  |  |
| 13 | همکارانم را تشویق میکنم تا به صورت ایمن کار کنند. |  |  |  |  |  |
| 14 | برای بهبود ایمنی محیط کارم تلاش زیادی می­کنم. |  |  |  |  |  |
| 15 | بطور فعال در جلسات ایمنی شرکت می کنم. | هرگز | به ندرت | گاهی | اغلب | همیشه |
| 16 | مسائل ایمنی قابل توجه را به مدیریت اطلاع می­دهم. |  |  |  |  |  |
| 17 | وقتی همکارانم در شرایط خطرناک کار می­کنند، به آنها کمک می­کنم. |  |  |  |  |  |
| 18 | طوری عمل می­کنم که برنامه های ایمنی در داخل شرکت ترویج و ارتقاء یابد. |  |  |  |  |  |
| 19 | به طور داوطلبانه فعالیت­هایی را انجام می­دهم که به بهبود ایمنی در محیط کارم کمک می­کند. |  |  |  |  |  |
| 20 | در جلسات ایمنی نکات مورد نظرم را ارائه داده و بیان می­کنم. |  |  |  |  |  |
| 21 | برای انجام یک کارخاص درمورد خطرات آن،اطلاعاتی را ازسرپرست ایمنی یا همکاران درخواست می­کنم |  |  |  |  |  |
| 22 | اقدامات اصلاحی یا بهسازی مربوط به ایمنی را به سرپرست یا مسئول ایمنی پیشنهاد می­کنم. |  |  |  |  |  |
| 23 | وضعیت خطرناک را به سرپرست یا نماینده ایمنی گزارش می­دهم. |  |  |  |  |  |

| \|  \|  \|  \| \| --- \| --- \| --- \| |  |
| --- | --- | --- | --- | --- |
